# Factors associated with the choice of supplementary hospital insurance in Switzerland – an analysis of the Swiss Health Survey

**DOI:** 10.1186/s12913-023-09221-0

**Published:** 2023-03-16

**Authors:** Szilvia Altwicker-Hámori, Michael Stucki

**Affiliations:** 1 Institute of Public Health, School of Health Sciences, ZHAW Zurich University of Applied Sciences, Katharina-Sulzer-Platz 9, Winterthur, 8400 Switzerland; 2 Winterthur Institute of Health Economics, School of Management and Law, ZHAW Zurich University of Applied Sciences, Gertrudstrasse 15, Winterthur, 8401 Switzerland

**Keywords:** Supplementary hospital insurance, Inpatient care, Switzerland, Probit, Swiss Health Survey

## Abstract

**Background:**

Switzerland has universal coverage via mandatory health insurance that covers a generous basket of health services. In addition to the basic coverage, the insured can buy supplementary insurance for the inpatient sector. Supplementary hospital insurance in Switzerland provides additional services during inpatient stays. Little is known about which factors are associated with the choice of semi-private and private hospital insurances. However, this is of importance to policy makers and the insured population, who might be concerned about a “two-class” inpatient care system. Therefore, the aim of the paper was to explore the factors associated with supplementary hospital insurance enrolment in Switzerland.

**Methods:**

We used the five most recent waves of the representative Swiss Health Survey (1997, 2002, 2007, 2012, 2017) to explore which factors are associated with supplementary hospital insurance enrolment in adults aged 25 or older. We estimated the same probit model for all five surveys waves and computed average marginal effects.

**Results:**

Our study shows that in all cross-sections the likelihood of enrolling in supplementary hospital insurance increased with higher age, education, household income and was higher for people with a strong preference for unrestricted choice of a specialist and with a higher-than-default deductible choice. The likelihood of supplementary hospital insurance enrolment was lower for the unemployed relative to their inactive counterparts and those living in rural areas relative to comparable urban residents. Ever-smoker status was not statistically significantly associated with supplementary hospital insurance choice. However, our findings indicated differences in estimates over the years regarding demographic as well as insurance-related variables. For example, women were more likely to choose supplementary hospital insurance than comparable men in earlier years.

**Conclusion:**

Most importantly, our results indicate that factors related to socioeconomic status – such as education, labour market status, and income – consistently show significant associations with the probability of having supplementary hospital insurance for the entire study period, as opposed to demographic variables – such as nationality and sex.

## Background

Switzerland introduced universal health coverage by enacting the Federal Law on Health Insurance (KVG) in 1996. Enrolment in the basic health insurance with one of about 50 private, non-profit companies is mandatory and no individual may be declined coverage. The premiums are community-rated and do not depend on income or the risk of falling ill. The average premium in 2017 amounted to CHF 4,224 per year for adults above the age of 26; representing a 109% increase relative to 1997 [1]. There are lower premiums for individuals who opt for an insurance model which restricts the freedom of choosing the health care provider (e.g., health maintenance organization or family doctor models) or which includes a higher-than-default yearly deductible (e.g., a maximum of CHF 2,500, as opposed to the default of CHF 300 in 2017). On top of the generous benefits package guaranteed by the mandatory health insurance [2], the insured can buy insurance for supplementary services. The latter may be provided by the same companies but are regulated outside of the social health insurance system. The share of private insurance in percent of total health care spending decreased over time: from 10.6% in 1995 to 8.5% in 2010, and 6.8% in 2017 [3].

In many countries with universal coverage, some form of voluntary private insurance is available (e.g., in most European countries [4]). Private health insurances can serve different functions: they can either cover individuals not covered by a mandatory social health insurance or provide supplementary coverage for services not covered by the mandatory health insurance, including the co-payments foreseen in social health insurance. In Switzerland, coverage of co-payments and deductibles is not allowed. There are, however, different types of supplementary insurances, including coverage of complementary outpatient care, dental services, prevention and health promotion measures, and services abroad. Supplementary insurance is most relevant for the coverage of supplementary services during inpatient stays at hospitals, also referred to as supplementary hospital insurance. These schemes are the focus of this study.

Supplementary hospital insurance is offered either as a semi-private or a private scheme. The main benefits of these insurances are higher standard accommodation (in double rooms for semi-privately insured and single rooms for privately insured), quicker access to treatments, and surgery performed by chief surgeons. Because there are hardly any waiting times in inpatient care in Switzerland [5], the main purpose of these insurance plans is higher comfort during inpatient stays. Health insurers can – in contrast to mandatory health insurance – refuse enrolment based on pre-existing medical conditions or age and may set risk-rated premiums. There is no publicly available data on the average supplementary insurance premium. According to comparis.ch, an online platform to compare insurance contracts, a 50-year-old new male enrollee living in the biggest canton, the canton of Zurich, would face costs of approximately CHF 1800–6600 yearly for a private hospital insurance in 2023. The respective figures for a 50-year-old female enrollee would be CHF 2100–6600 yearly.

The number of people enrolled in private hospital insurance schemes in Switzerland has decreased after the implementation of the Federal Law on Health Insurance in 1996 [6], but very little is known about the factors associated with the uptake of private hospital insurance in Switzerland. A recent descriptive analysis of data from the Swiss Health Survey (SHS) for 2017, i.e., one wave of the same data source we are using in our study, shows that the share of respondents with a semi-private or private hospital insurance increased with age and education, and it was higher in urban areas [6]. However, this study did not carry out multivariate analyses to assess the varying probability of supplementary hospital insurance uptake across different demographic and socioeconomic groups – an important equality concern.

There are several international studies investigating the factors associated with buying voluntary insurance in addition to basic social health insurance [7–16]. The existing evidence suggests that the uptake of voluntary health insurance is mostly related to demographic and socioeconomic factors [17]. The review by Kiil et al. (2012) covered many countries world-wide (though not Switzerland) and found that enrolment in voluntary health insurance increases with age (or increases up to a certain age and decreases after), urban residence, income, education, and employment; results were mixed for sex (with most studies finding higher probabilities among women).

Three studies used the Survey of Health, Ageing and Retirement in Europe (SHARE) to analyse the determinants of the demand for voluntary health insurance in a number of European countries offering different forms of insurance [7, 13, 16]. The SHARE does not distinguish between different types, i.e., supplementary insurances like the private hospital insurance or dental insurance as well as complementary coverage of co-payments were included. Bolin et al. (2010) focused on whether better health decreases the probability of enrolment in any voluntary health insurance [7]. Their study pooled ten European countries (including Switzerland) and showed that people in better health are more likely to buy more health insurance. Paccagnella et al. (2013) included 11 countries (including Switzerland) in their study and estimated one model for each country separately. The authors concluded that voluntary health insurance plans were mainly bought by people who are in better health, have higher income and higher education [13]. Moreover, they found that cognitive ability was positively associated with the probability of voluntary health insurance in most countries (the effect for Switzerland was, however, not significant). The recent study by Tavares (2020) pooled data from 18 countries and found that individuals who are more satisfied with the basic coverage in the health care system are more likely to opt for voluntary private health insurance [16].

Belgium has a private hospital insurance scheme that shares some features with the Swiss one. The insurance covers extra charges by the hospitals for single rooms and other resources used in treatment. Schokkaert et al. (2010) used data from the cross-sectional Health Interview Survey from 2001 and investigated the determinants of supplementary hospital insurance enrolment [15]. They found that the probability of enrolment mainly depends on socioeconomic inequalities; it increases with age, income, and education. Furthermore, unemployed individuals and those who considered their self-assessed health to be poor, good or very good were less likely to buy supplementary hospital insurance compared to those in intermediate health.

Generalising the results from other countries to Switzerland might be misleading because of the considerable cross-country differences in health care systems and health insurance systems. Therefore, this study’s contribution is twofold. First, we investigate the factors associated with supplementary hospital insurance enrolment in Switzerland for each SHS wave separately and are thus able to detect changes in the associations over time. Second, we include a high number of factors possibly related to the choice for supplementary hospital insurance. To the best of our knowledge, there is currently no study assessing these factors in a multivariate analysis for Switzerland.

## Methods

### Data

We used repeated cross-sectional data from the SHS. The SHS has been conducted by the Federal Statistical Office (FSO) since 1992 every five years and includes data collected in a telephone interview and a follow-up written questionnaire in each wave [18]. The SHS covers a representative sample of the population above 15 years and contains information about health status, health behaviour, health care utilization, insurance status as well as demographic and socioeconomic information.

#### Sample selection

**Fig. 1:**
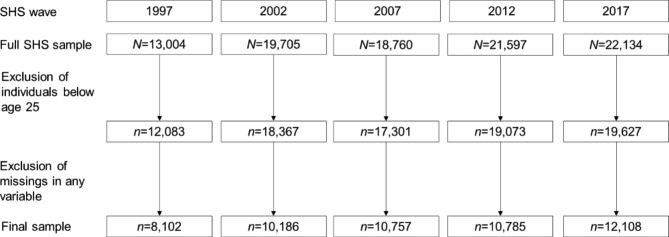
Sample selection

A number of steps were taken to arrive at our final cross-sectional samples. First, the 1992 SHS was omitted from our analysis as the Federal Law on Health Insurance was only implemented in 1996, which represented a change in the insurance market structure. Second, we included only adults aged 25 or older. The reasons for excluding younger respondents were twofold: first, many of these individuals are possibly still in full-time education and might thus have a different choice set; second, the decision about supplementary hospital insurance enrolment in young people is likely to depend on the parents’ decision rather than their own. Finally, observations with missing values in the dependent and independent variables were excluded. The final yearly sample sizes ranged from 8,102 in 1997 to 12,108 in 2017 (for further details on the sample selection by year see Fig. 1).

#### Variables

##### *Dependent variable*

The SHS contains information about the insurance coverage in case of an inpatient stay. The respective question is: “How are you insured when you have to go to the hospital?”. Between 1997 and 2007 the aforementioned information was collected via telephone interviews and included the following options: “general ward”, “semi-private ward”, “private ward”, “I do not know”. Starting 2012, the information was collected in the paper-and-pencil questionnaire and included one more answer option: “other model”. However, only a small minority chose this option (i.e., 182 individuals in our sample in 2012 and 211 in 2017, respectively; and there was no further information on what these other models cover in case of inpatient care).

We used the answer options to generate a binary variable differentiating between “private” and “general”. The “private” category merged respondents who *explicitly* stated to have semi- or private ward coverage. The “general” category merged respondents with “general ward”, i.e., no supplementary coverage, and those who reported “other model” (relevant only in 2012 and 2017). We dropped respondents from the sample if they answered “I don’t know” or if the answer was missing in the written questionnaire (only relevant in 2012 and 2017). In the remainder of the paper, we use “private” and “supplementary” hospital insurance interchangeably.

##### *Independent variables*

We included a variety of independent variables: demographic variables (sex, age, nationality, marital status, and number of children in same household); socioeconomic variables (education level, labour market status, and household income), regional indicators (language region and rural/urban residence); one variable capturing health behaviour (ever-smoker or not) as well as variables concerning the mandatory insurance model the respondent was enrolled in. Individuals can choose contracts in the mandatory health insurance differentiated along two dimensions: first, the degree of freedom in the choice of their provider (either non-restricted access provided in the standard model or restricted access in a managed care model); second, the yearly deductible. Finally, we included the respondent’s preference for free choice of specialists. We used the aforementioned regional variables for two reasons. First, the analyses of voting results as well as survey data repeatedly reveal strong differences in political attitudes and preferences by language region in Switzerland [19]. Second, both Swiss and international evidence implies that enrolment in voluntary health insurance is higher for those living in urban areas. The covariates are described in detail in Table 1.

**Table 1:** Description of independent variables

Variable	Description	Unit / Levels
Sex	Sex	0 = male 1 = female
Age	Five-year age groups	25–29; …; 80+
Nationality	Nationality	0 = Swiss 1 = Non-Swiss
Marital status	Marital status	0 = not married/single, divorced, and widowed 1 = married or registered partnership
Number of children in same household	Number of children (< 18 years) living in the same household	0; 1; 2; 3+
Education	Level of formal schooling	1 = compulsory 2 = secondary 3 = tertiary
Labour market status	Labour market status	1 = inactive 2 = unemployed 3 = employed
Household income^,1^	Household net income per month, quintile	1st-5th quintile
Language region	Language of the respondent’s residence	0 = German and Romansh 1 = French 2 = Italian
Rural area	Urbanicity of the respondent’s residence	0 = urban 1 = rural
Ever smoker	Respondent has ever smoked	0 = no 1 = yes
Deductible^,2^	Yearly deductible in the basic health insurance	0 = standard/lowest (default) 1 = choice deductible higher than default
Standard insurance model^,3^	Standard model in the mandatory health insurance (without restricted access to providers)	0 = no 1 = yes
Preference for free specialist choice	Importance of having the possibility to choose specialist without restrictions	0 = rather not important or not important 1 = important or very important

^1^ *Compulsory education corresponds to nine years of schooling in Switzerland, secondary education corresponds to either high school or vocational training, and tertiary education corresponds to a university degree (including universities of applied sciences and Federal Institutes of Technology)*

^2^ *Income refers to the self-reported income of all members of the same household, after deducting social security contributions and adding/deducting alimonies. For single-household individuals, we replaced this variable by the self-reported personal income variable.*

^2^ *The available deductible levels were changed multiple times during the study period. We recoded the information in the survey as displayed here to make it comparable across all years under analysis.*

^3^ *We differentiated only between the standard and any other model type, including health maintenance organizations (HMOs), preferred providers lists, and telemedicine models.*

### Regression models

The aim of the analyses was to identify factors associated with the choice of supplementary hospital insurance. We therefore estimated binary probit models. In all the analyses, we applied survey weights provided in the SHS. Variance inflation factor (VIF) was used to assess multicollinearity; there was no indication of multicollinearity. Results of the probit models are presented as average marginal effects (AMEs). The criterion for statistical significance was set at *p* < 0.10.

## Results

### Summary statistics

The share of those aged 25 and above with supplementary hospital insurance decreased over time: it was as high as 40% in 1997 and decreased continually to 28% in 2017 (2002: 33%, 2007: 29%, 2012: 28%).

Table 2 shows summary statistics for all the explanatory variables by supplementary hospital insurance status and survey year. Starting with the most recent wave (2017), the proportion of women was slightly higher in the subsample with general insurance than in the privately insured subsample (50% and 47%, respectively). The share of age groups above age 55 was higher in those with private insurance compared to those with general insurance (52% versus 38%). The share of non-Swiss respondents was lower in the privately insured group than in the group with general insurance (19% and 23%, respectively). Respondents with private insurance were more often married (64% versus 58%) and were living in smaller families on average (77% and 69% reported no child living in the same household, respectively).

**Table 2:** Summary statistics for independent variables, by supplementary hospital insurance status and survey year

	General 1997	Private 1997	General 2002	Private 2002	General 2007	Private 2007	General 2012	Private 2012	General 2017	Private 2017
Female (%)	47.5	51.9	50.5	52.0	49.0	49.5	47.5	48.6	50.2	46.9
Age groups (%)										
25–29	13.0	6.2	8.9	5.2	10.2	4.1	8.1	4.6	7.8	5.3
30–34	15.9	10.1	15.6	8.3	12.2	6.1	10.5	6.5	11.2	6.5
35–39	13.5	12.2	15.0	8.8	13.6	7.4	10.4	6.1	10.7	8.0
40–44	10.4	11.7	12.0	10.6	14.0	11.8	12.4	9.0	10.0	8.9
45–49	8.6	12.5	9.6	12.5	11.1	10.1	13.8	13.5	10.9	8.6
50–54	7.3	12.6	8.2	12.7	9.4	11.4	10.1	9.5	11.5	11.1
55–59	6.0	9.5	6.9	13.0	7.0	10.9	8.1	8.8	8.9	9.1
60–64	5.9	8.1	5.8	8.7	6.6	12.7	7.0	9.8	8.2	9.9
65–69	5.8	5.5	5.2	6.8	5.1	8.8	6.8	11.0	6.3	9.8
70–74	6.1	6.3	6.3	6.2	4.3	6.3	4.5	7.5	5.4	9.1
75–79	4.0	2.9	3.8	4.4	3.5	5.6	4.4	6.1	4.7	7.2
80+	3.6	2.5	2.7	2.7	3.0	4.8	3.9	7.6	4.4	6.5
Non-Swiss (% yes)	20.5	11.5	19.7	12.3	18.7	11.6	21.9	16.8	23.2	19.1
Rural (% yes)	33.1	25.6	34.1	25.0	28.7	19.8	27.6	19.7	28.1	19.5
Marital status (% married)	65.5	71.0	69.8	72.1	61.8	66.7	59.2	63.3	58.2	63.5
Number of children in household (number)										
0	64.1	67.5	59.9	72.1	63.8	76.7	61.1	70.8	69.4	77.3
1	12.6	12.7	14.8	11.4	14.9	9.3	14.9	11.7	12.4	9.9
2	16.4	15.1	18.5	12.7	15.6	10.7	17.2	13.1	14.0	10.1
3+	6.8	4.7	6.8	3.8	5.7	3.3	6.7	4.3	4.2	2.7
Language regions (%)										
German/Romansh	69.6	69.6	68.8	71.0	72.3	74.0	73.3	71.4	73.8	72.3
French	26.2	24.6	26.9	23.2	23.7	21.7	21.8	24.1	21.9	23.1
Italian	4.2	5.9	4.4	5.9	4.0	4.3	4.9	4.5	4.3	4.6
Education (%)										
Compulsory school	24.3	10.8	19.8	7.8	15.7	7.3	14.2	7.3	13.8	5.8
Secondary school	58.2	64.3	63.9	66.2	58.7	53.4	54.0	47.5	49.6	42.3
Tertiary education	17.5	25.0	16.3	26.0	25.6	39.4	31.8	45.2	36.6	51.9
Labour market status (%)										
Inactive	28.5	29.0	26.4	30.7	22.0	30.7	25.1	34.7	26.0	33.8
Unemployed	3.4	1.5	1.7	0.8	1.9	1.3	2.0	0.6	2.5	1.0
Employed	68.0	69.5	71.9	68.5	76.1	68.0	72.9	64.7	71.6	65.1
Household income (%)										
1st quintile	18.9	7.6	15.9	10.0	14.7	8.3	19.4	12.3	21.7	11.5
2nd quintile	20.6	12.5	20.8	11.5	20.0	13.5	24.2	16.5	22.4	16.0
3rd quintile	25.8	21.6	22.1	16.0	26.9	18.4	23.8	18.7	20.6	19.3
4th quintile	20.0	24.2	23.8	22.4	18.4	18.5	16.6	19.2	21.1	21.9
5th quintile	14.7	34.1	17.4	40.1	19.9	41.3	16.0	33.2	14.3	31.2
Ever smoker (% yes)	54.9	54.3	51.8	53.4	51.3	53.1	54.0	50.0	52.3	51.0
Choice deductible higher than default (% yes)	52.5	62.8	61.3	68.2	64.0	65.3	61.7	63.8	61.0	64.8
Standard insurance model (% yes)	96.7	94.9	93.9	92.3	82.8	83.8	51.8	55.3	43.8	48.4
Preference for free specialist choice (% important/very important)	68.5	80.8	67.3	83.9	66.0	83.3	67.4	87.6	68.3	88.5
Observations	4,933	3,169	6,723	3,463	7,451	3,306	7,619	3,166	8,654	3,454

Differences in private insurance enrolment were substantial with respect to education: while the share of those with tertiary education was only approximately 37% in the group with general insurance, 52% of those privately insured obtained a tertiary degree. In terms of labour market status, the subsample of privately insured contained less employed individuals than the subsample with general insurance (65% and 72%, respectively). Among those with a private insurance, the share of people in the top quintile with respect to household income was largest (31%); the respective number was merely 14% for those with general insurance.

The share of respondents residing in rural regions was lower in those privately insured (20% versus 28%). On the contrary, the distribution in terms of language region of residence and smoker status was approximately the same in the two groups.

Those with a private insurance more frequently opted for a higher deductible (65% versus 61%) and were more often insured in the standard insurance model in the mandatory insurance (48% versus 44%). The share of respondents who indicated that choosing their specialist freely is important for them was substantially higher among those with private insurance (89% versus 68%).

There were differences over the study period in almost all explanatory variables. For instance, a shift in the educational composition could be observed: 42% and 64% of the privately insured obtained a secondary degree in 2017 and 1997, respectively; the corresponding numbers for those with general insurance were 50% and 58% in the respective years.

### Regression results

Table 3 displays probit estimation results (AMEs and corresponding standard errors) for each year. Starting with the most recent SHS wave (2017), the oldest age groups were consistently more likely to have private insurance relative to their counterparts aged 25–29 years. The AME of having private insurance amounted to approximately 6%-points in those aged 55–59; it increased up to 28%-points for those aged 80 years and over. Compared to individuals without children living in the same household, their counterparts with two children living in the same household had a lower probability of having private insurance (about − 6%-points).

**Table 3:** Average marginal effect on probability of supplementary hospital insurance (probit estimation)

	1997		2002		2007		2012		2017	
	AME	SE	AME	SE	AME	SE	AME	SE	AME	SE
Female	0.0950^***^	(0.0125)	0.0590^***^	(0.0116)	0.0520^***^	(0.0104)	0.0374^***^	(0.0106)	0.0093	(0.0096)
Age group (base: 25–29)										
30–34	0.0504^**^	(0.0199)	-0.0217	(0.0242)	0.0290	(0.0197)	0.0079	(0.0259)	-0.0211	(0.0235)
35–39	0.1185^***^	(0.0220)	0.0148	(0.0248)	0.0277	(0.0188)	0.0024	(0.0252)	0.0198	(0.0242)
40–44	0.1525^***^	(0.0236)	0.0696^***^	(0.0258)	0.1020^***^	(0.0206)	0.0187	(0.0244)	0.0403^*^	(0.0241)
45–49	0.1897^***^	(0.0249)	0.1347^***^	(0.0273)	0.1153^***^	(0.0221)	0.0751^***^	(0.0246)	0.0086	(0.0227)
50–54	0.2439^***^	(0.0255)	0.1541^***^	(0.0267)	0.1602^***^	(0.0228)	0.0620^**^	(0.0243)	0.0363	(0.0221)
55–59	0.2789^***^	(0.0276)	0.2153^***^	(0.0281)	0.2121^***^	(0.0243)	0.1108^***^	(0.0256)	0.0631^***^	(0.0242)
60–64	0.2756^***^	(0.0278)	0.1814^***^	(0.0290)	0.2911^***^	(0.0253)	0.1864^***^	(0.0270)	0.1281^***^	(0.0245)
65–69	0.2179^***^	(0.0305)	0.1699^***^	(0.0311)	0.3004^***^	(0.0286)	0.2470^***^	(0.0292)	0.1999^***^	(0.0276)
70–74	0.2773^***^	(0.0313)	0.1518^***^	(0.0322)	0.2969^***^	(0.0304)	0.2834^***^	(0.0323)	0.2463^***^	(0.0287)
75–79	0.2117^***^	(0.0390)	0.1718^***^	(0.0358)	0.3455^***^	(0.0327)	0.2666^***^	(0.0361)	0.2637^***^	(0.0315)
80+	0.2001^***^	(0.0391)	0.1592^***^	(0.0398)	0.3743^***^	(0.0347)	0.3413^***^	(0.0372)	0.2763^***^	(0.0331)
Non-Swiss	-0.0831^***^	(0.0170)	-0.0418^**^	(0.0179)	-0.0441^***^	(0.0166)	0.0041	(0.0157)	0.0092	(0.0131)
Language region (base: German/Romansh)										
French	-0.0058	(0.0131)	-0.0269^**^	(0.0122)	-0.0155	(0.0109)	0.0130	(0.0117)	0.0139	(0.0105)
Italian	0.1173^***^	(0.0211)	0.0742^***^	(0.0193)	0.0389^**^	(0.0185)	0.0219	(0.0184)	0.0502^***^	(0.0173)
Rural area	-0.0595^***^	(0.0125)	-0.0521^***^	(0.0116)	-0.0584^***^	(0.0112)	-0.0371^***^	(0.0124)	-0.0579^***^	(0.0105)
Married	-0.0102	(0.0137)	0.0033	(0.0124)	-0.0062	(0.0117)	-0.0114	(0.0122)	-0.0047	(0.0110)
Number of children in household (base: 0)										
1	0.0176	(0.0207)	-0.0420^**^	(0.0191)	-0.0562^***^	(0.0172)	-0.0152	(0.0177)	-0.0257	(0.0167)
2	0.0022	(0.0198)	-0.0671^***^	(0.0189)	-0.0286	(0.0180)	-0.0121	(0.0188)	-0.0557^***^	(0.0159)
3 or more	-0.0701^***^	(0.0265)	-0.1012^***^	(0.0243)	-0.0514^**^	(0.0256)	-0.0419	(0.0295)	-0.0601^**^	(0.0247)
Education (base: Compulsory school)										
Secondary school	0.1551^***^	(0.0152)	0.1493^***^	(0.0143)	0.1099^***^	(0.0131)	0.0821^***^	(0.0162)	0.0820^***^	(0.0150)
University degree	0.1821^***^	(0.0200)	0.2081^***^	(0.0195)	0.1950^***^	(0.0162)	0.1555^***^	(0.0184)	0.1496^***^	(0.0165)
Labour market status (base: inactive)										
Unemployed	-0.1260^***^	(0.0390)	-0.1203^**^	(0.0475)	-0.0023	(0.0490)	-0.1306^***^	(0.0384)	-0.0860^**^	(0.0388)
Employed	-0.0422^**^	(0.0171)	-0.0520^***^	(0.0158)	-0.0356^**^	(0.0158)	-0.0384^**^	(0.0169)	-0.0312^**^	(0.0150)
Household income (base: 1st quintile)										
2nd quintile	0.1195^***^	(0.0169)	-0.0083	(0.0159)	0.0697^***^	(0.0136)	0.0685^***^	(0.0138)	0.0763^***^	(0.0130)
3rd quintile	0.1909^***^	(0.0173)	0.0612^***^	(0.0173)	0.0997^***^	(0.0140)	0.1088^***^	(0.0148)	0.1438^***^	(0.0143)
4th quintile	0.2648^***^	(0.0193)	0.1106^***^	(0.0176)	0.1771^***^	(0.0167)	0.1835^***^	(0.0169)	0.1767^***^	(0.0146)
5th quintile	0.3919^***^	(0.0204)	0.2718^***^	(0.0192)	0.3027^***^	(0.0168)	0.2981^***^	(0.0180)	0.3154^***^	(0.0164)
Ever smoker	-0.0047	(0.0116)	0.0101	(0.0106)	0.0119	(0.0100)	-0.0077	(0.0102)	0.0064	(0.0091)
Choice deductible higher than default	0.0699^***^	(0.0117)	0.0540^***^	(0.0110)	0.0349^***^	(0.0107)	0.0419^***^	(0.0107)	0.0522^***^	(0.0097)
Standard insurance model	-0.0944^***^	(0.0309)	-0.0530^**^	(0.0217)	-0.0112	(0.0141)	0.0029	(0.0105)	0.0145	(0.0095)
Preference for free specialist choice important/very important	0.1319^***^	(0.0128)	0.1607^***^	(0.0114)	0.1496^***^	(0.0103)	0.1837^***^	(0.0107)	0.1868^***^	(0.0097)
Observations	8,102		10,186		10,757		10,785		12,108	

AME: Average marginal effect; SE: Standard error;^*^p < 0.10, ^**^p < 0.05, ^***^p < 0.01

Some of the largest AMEs were found for socioeconomic status. The probability of having private insurance increased significantly for those with higher education (i.e., approximately 8%-points and 15%-points for those with a secondary and tertiary degree, respectively, compared to their counterparts with only compulsory education). Both the unemployed and employed respondents were less likely to have private insurance relative to their inactive counterparts (around − 9%-points and − 3%-points, respectively). The AMEs were increasing with household income: the AME amounted to about 8%-points at the 2nd quantile and to 32%-points at the top quantile compared to their counterparts in the lowest quintile.

Respondents from the Italian-speaking region in Switzerland had a higher probability of private insurance (around 5%-points) compared to their counterparts residing in the German-speaking region, while those residing in rural areas had a lower probability (about − 6%-points) relative to their counterparts residing in urban areas.

Individuals with a higher-than-default deductible in the mandatory health insurance were more likely to have supplementary hospital insurance (approximately 5%-points) than their counterparts with the default level. Indicating strong preference for unrestricted choice of specialists increased the probability of having private insurance by around 19%-points.

Sex, nationality, marital status, ever-smoking status, and being enrolled in the standard model in the mandatory health insurance showed no statistically significant associations with private insurance in 2017.

The estimation results showed numerous noteworthy differences over the study period. First, women in all previous years showed a higher likelihood of having private insurance relative to their male counterparts; however, the respective AMEs decreased over the study period (i.e., the AME amounted to around 10%-points in 1997 and merely 4%-points in 2012). Second, the AMEs were positive and statistically significant for all age groups in 1997; in the later years, this only held for increasingly older age groups. Third, up until 2007, foreign nationals were less likely to be privately insured relative to their Swiss counterparts; here, too, a decreasing trend was observable (e.g., AME of approximately − 8%-points and − 4%-points in 1997 and 2007, respectively). Starting from 2012, the effect was not statistically significant. Fourth, we found a general negative association between the number of children living in the same household and the likelihood of having private insurance over the study period – albeit differences in statistical significance. Finally, a statistically negative association between choosing a standard insurance model and private insurance could only be observed in the beginning of the study period (i.e., AME of approximately − 9%-points and − 5%-points in 1997 and 2002, respectively).

As a robustness check, we estimated the regression models with all observations including those with missing values in the independent variables (as separate categories) and the results remained robust.

## Discussion

### Main findings

Despite the relevance of supplementary hospital insurance in the financing of the inpatient sector of the Swiss health care system, the factors associated with the choice of this scheme have not been studied in detail so far. Our aim was to close this gap by investigating the relationship between demographic, socioeconomic, health-related, regional, and other variables related to the insurance coverage in the mandatory health insurance in adults aged 25 and older living in Switzerland. We carried out repeated cross-sectional analysis using the five most recent waves of the SHS (1997 to 2017).

Our descriptive statistics showed a decreasing time trend in supplementary hospital insurance. This may be linked to insurance-related factors such as the premium or the attractiveness/additional benefits of the insurance coverage compared to the mandatory health insurance. The attractiveness of private insurance has decreased for mainly two reasons. First, its marginal benefit has been decreasing: many treatments are shifted to the outpatient sector thanks to technological progress [20, 21]; the standard in the inpatient setting has increased even for those with only basic insurance (e.g., the new hospital financing reform implemented in 2012 allows everybody to choose their hospital freely); many new hospitals offer double or single rooms even to patients without any private insurance coverage [22]. Second, premiums have been rising substantially, which was identified as the number one reason to quit a private insurance [19]. According to numbers by the Federal Office of Public Health, premiums for private and semi-private increased by 55.1% and 46.1%, respectively, between 1999 and 2017 [23].

Our estimation results for the most recent SHS wave (2017) showed that the probability of supplementary hospital insurance increases with higher age (i.e., above the age of 55). This is likely to be demand-driven as older people have a higher risk of falling ill and needing inpatient care. The higher probabilities in older age groups are possibly linked to a cohort effect. Before the introduction of the Federal Law on Health Insurance (KVG) in 1996, it was more common to have a private hospital insurance. Those enrolled in private hospital insurance plans before 1996 were likely to keep the insurance even after there was a basic health insurance for everybody [6]. Interestingly, the other demographic variables (sex, nationality, marital status) were not statistically significantly associated with private insurance; neither was ever-smoker status.

Some of the variables we included as covariates reflect socioeconomic status, such as household income and education. The effects for household income followed a simple pattern. The probability of private insurance enrolment increased with household income; in 2017, it was about 32%-points higher for those from the top quintile compared to those from the bottom quintile. Higher education was associated with an increased likelihood of having supplementary hospital insurance. As this effect was found after controlling for income, it might reflect more risk aversion in those with more than compulsory schooling [24] or might be driven by better health status in the better educated, which increases the chances of obtaining any supplementary hospital insurance. Education might also be linked to health literacy. Previous literature has shown that better cognitive ability is associated with a higher probability of choosing a voluntary health insurance (of any type) for 11 European countries including Switzerland and its neighbours Germany, France, Italy, and Austria [13]. Even though we were not able to explicitly account for cognitive ability, it is important to keep in mind that individuals may differ in their abilities to evaluate costs and benefits associated with a supplementary hospital insurance contract.

Out of the insurance-model characteristics, the preference for unrestricted choice of specialists had the strongest associations with the probability of having any supplementary hospital insurance. Since a reform in 2012, Swiss residents have access to all hospitals listed by any canton (region), even if it is located outside the canton of their residence and if they have no private hospital insurance. However, a private insurance is still required to get treatment by chief surgeons. Therefore, the choice of the professional may be driving the results.

Overall, our research findings for 2017 confirm results from previous international literature investigating the factors related to holding supplementary health insurance in that socioeconomic variables were found to be the most important explanatory variables [13, 15, 17]Although the estimation results of the repeated cross-sectional analyses were consistent in terms of the socioeconomic variables, they pointed to differences in demographic characteristics including sex, age, number of children living in the same households, and nationality. These differences call for the analysis of the most recent SHS waves (upon availability) in order to establish potential trends.

### Methodological considerations

The rich data from the SHS allowed for an extensive analysis of the factors associated with holding semi-private or private insurance in a representative sample of the Swiss population. More specifically, as opposed to the few previous studies investigating voluntary insurance choice in general, we could focus on private hospital insurance, could rely on repeated cross-sections as well as a broader range of covariates.

Our study has several limitations, however. First, the use of repeated cross-sectional data did not allow us to track individuals and their coverage decisions over time. We would need longitudinal data to perform such an analysis. Our data did not allow for the identification of factors associated with the decision to *change* private insurance or *enrol* for the first time, but it only showed the variables linked to *having* supplementary coverage, independently of when the insurance contract was signed. In 2002, the SHS included a question about whether the respondent changed his or her private insurance and in which direction the change was made. However, the number of observations was low in this cross-section (*n* = 369) and the question was not repeated in later waves. There is room for further research in this area, as both the reasons for changing private insurance and the characteristics of those changing private insurance is understudied in Switzerland.

Second, the present analysis does not account for cohort effects, a highly relevant issue. Therefore, an age-period-cohort (APC) analysis would be desirable in future research. Third, the widely documented issue of potential income under- and misreporting in surveys applies to our study [25]. To this end, further analyses using matched register and survey information regarding income would be valuable. Fourth, conducting multiple imputations as an alternative to deal with missing values could be addressed in future research. Fourth, our results must be interpreted keeping in mind that we could not account for the fact that health insurers may refuse enrolment based on pre-existing medical conditions. Incorporating data on refusals or data on previous health conditions relevant for refusals would be necessary to analyse potential selection bias. Finally, it is important to stress that the effects found in the present paper cannot be interpreted in a causal way. Nevertheless, the associations call attention to factors that should be taken into account in “equality” considerations.

Overcoming the limitations described above is therefore the focus of our future research. Most importantly, the use of longitudinal data and APC models would be highly relevant.

## Conclusion

This study analysed the factors associated with having a supplementary hospital insurance (either semi-private or private) in Switzerland. It is the first study using multiple waves of the SHS (1997–2017) and accounting for a broad range of potential factors associated with supplementary hospital insurance enrolment. Most importantly, we found consistent positive associations between numerous socioeconomic variables (e.g., income, education) and supplementary hospital insurance enrolment. Our findings are thus consistent with the recent empirical evidence indicating that supplementary hospital insurance is a “luxury good” [6]. However, our results point to important differences in the estimation results regarding demographic characteristics (sex, age, number of children living in the same households, and nationality) over the study period. This in turn highlights the value in estimating repeated cross-sections in order to identify groups with differences in access to supplementary hospital insurance.

## Data Availability

The data that support the findings of this study are available from the Swiss Federal Statistical Office (FSO), but restrictions apply to the availability of these data, which were used under license for the current study, and so are not publicly available. Request for access can be sent to sgb@bfs.admin.ch.

## Abbreviations

AME: Average marginal effect
APC: age-period-cohort
FSO: Federal Statistical Office
HMO: Health maintenance organisation
KVG: Federal Law on Health Insurance (Krankenversicherungsgesetz)
SE: Standard error
SHARE: Survey of Health, Ageing and Retirement in Europe
SHS: Swiss Health Survey
